# EDS1 modules as two-tiered receptor complexes for TIR-catalyzed signaling molecules to activate plant immunity

**DOI:** 10.1007/s44154-022-00056-z

**Published:** 2022-08-10

**Authors:** Jia Li, Xiaorong Tao

**Affiliations:** The Key Laboratory of Plant Immunity, Department of Plant Pathology, Nanjing Agricultural University, Nanjing, 210095 People’s Republic of China

**Keywords:** NLR receptor, Toll/Interleukin-1 receptor domain, EDS1, PAD4, SAG101, Signaling molecule, Plant immunity

## Abstract

Plant intracellular nucleotide-binding leucine-rich repeat (NLR) receptors with an N-terminal Toll/Interleukin-1 receptor (TIR) domain detect pathogen effectors to produce TIR-catalyzed signaling molecules for activation of plant immunity. Plant immune signaling by TIR-containing NLR (TNL) proteins converges on Enhanced Disease Susceptibility 1 (EDS1) and its direct partners Phytoalexin Deficient 4 (PAD4) or Senescence-Associated Gene 101 (SAG101). TNL signaling also require helper NLRs N requirement gene 1 (NRG1) and activated disease resistance 1 (ADR1). In two recent remarkable papers published in Science, the authors show that the TIR-containing proteins catalyze and produce two types of signaling molecules, ADPr-ATP/diADPR and pRib-AMP/ADP. Importantly, they demonstrate that EDS1-SAG101 and EDS1-PAD4 modules are the receptor complexes for ADPr-ATP/diADPRp and Rib-AMP/ADP, respectively, which allosterically promote EDS1-SAG101 interaction with NRG1 and EDS1-PAD4 interaction with ADR1. Thus, two different small molecules catalyzed by TIR-containing proteins selectively activate the downstream two distinct branches of EDS1-mediated immune signalings. These breakthrough studies significantly advance our understanding of TNL downstream signaling pathway.

Plants utilized both cell-surface and intracellular immune receptors to detect pathogen disturbance and initiate signaling cascades leading to host innate immunity. Nucleotide-binding leucine-rich repeat-containing (NLR) proteins are intracellular receptors with crucial roles in recognizing pathogen-delivered effectors and activating effector-triggered immunity (ETI). ETI is often associated with strong host defense responses and a localized cell death to restrict pathogen infection (Cui et al. 2015). Plant NLRs typically contain three major domains, including an N-terminal domain, a central NB-ARC domain (nucleotide-binding adaptor shared by Apaf-1, certain Resistance proteins, and CED-4), and a C-terminal leucine-rich repeat (LRR) domain. Based on their N-terminal domains, pathogen sensing NLRs are categorized into two major subclasses: CNL receptors with N-terminal coiled-coil (CC) domains and TNL receptors with Toll/interleukin-1 receptor (TIR) domainss. Direct or indirect recognition of pathogen effectors induces NLR oligomerization, forming a large NLR complex termed resistosome. Recently, three dimensional structures of resistosome from both CNL and TNL receptors have been resolved. The CNL resistosome from Arabidopsis ZAR1 forms pentamers and functions as a calcium-permeable cation channel to trigger cell death (Wang et al. 2019; Bi et al. 2021). The TNL resistosome from both Arabidopsis RPP1 and *Nicotiana benthamiana* ROQ1 form tetramers and function as nicotinamide adenine dinucleotide hydrolase (NADase) enzyme (Ma et al. 2020; Martin et al. 2020).

The TIR domain is an immune signaling module in many species. Recently, bacterial, plant and animal TIR domains were all found to possess NAD^ +^ -catalyzing enzymatic activities (Horsefield et al. 2019; Wan et al. 2019). Both cell death induction and NAD^+^ enzymatic activities of plant TIR domains depend on a putative catalytic glutamic acid that is conserved in bacterial TIR NADase and the mammalian SARM1 (sterile alpha and TIR motif containing 1) NADase, suggesting that the signaling role of TIR containing proteins require their NADase activity (Horsefield et al. 2019; Wan et al. 2019). Based on the cryo-EM structures of the RPP1 and ROQ1 resistosome (Ma et al. 2020; Martin et al. 2020), these TNLs formed tetramers upon recognizing the pathogen effector and tetramerization of TIR domains creates the active site for NADase. Thus, recognition of the pathogen effector enables TIR NADase activity and initiates the downstream immune signaling.

TNLs-mediated downstream plant immune signaling requires the conserved lipase-like protein Enhanced Disease Susceptibility 1 (EDS1) and its direct partners Phytoalexin Deficient 4 (PAD4) or Senescence-Associated Gene 101 (SAG101) (Lapin et al. 2019; Lapin et al. 2020). Moreover, TNLs also rely on helper NLRs to promote immunity and host cell death. N requirement gene 1 (NRG1) and activated disease resistance 1 (ADR1) are two sub-families of helper NLRs with their N-terminal CC domains homologous to Arabidopsis resistance to powdery mildew 8 (RPW8) and fungal and mammalian mixed lineage kinase cell death executors (MLKLs) which are known to function as cation channel (Peart et al. 2005; Collier et al. 2011; Mahdi et al. 2020). These helper NLRs are termed RNLs. Overexpression of NRG1 and ADR1 can cause cell death in *N. tabacum* (Collier et al. 2011). The structure of AtNRG1.1 CC_R_ domain was recently resolved and the activated AtADR1 and AtNRG1 were also found to function as calcium-permeable cation channels to trigger cell death (Jacob et al. 2021). In *N. benthamiana*, NRG1 functions downstream of EDS1 to confer TNL-mediated plant immunity and induces cell death (Qi et al. 2018). For TNL-mediated immunity in Arabidopsis, EDS1 forms heterodimers with PAD4. EDS1 uses the same surface to interact with SAG101 (Wagner et al. 2013). Recent studies have shown that the EDS1-PAD4 heterodimer co-function with RNL ADR1 and the EDS1-SAG101 heterodimer co-function with RNL NRG1 in TNL receptor-triggered immunity (Lapin et al. 2019; Sun et al. 2021). Upon effector recognition by TNL, the EDS1-SAG101 complex interacts with NRG1 and EDS1-PAD4 complex interacts with ADR1. In Arabidopsis, the EDS1-SAG101-NRG1 and EDS1-PAD4-ADR1 complexes are two distinct non-interchangeable signalling branches in ETI (Sun et al. 2021). The EDS1-SAG101-NRG1 branch mainly controls host cell death, while the EDS1-PAD4-ADR1 branch regulates host defense activation (Lapin et al. 2019). Thus, two functionally different signalling nodes, EDS1-SAG101-NRG1 and EDS1-PAD4-ADR1, mediate defences downstream of TNL receptor activation. These findings formed a model that the small molecule products generated by TNL NADase activities are intercepted by EDS1 heterodimers for the activation of two helper NLR subclasses. However, the molecular mechanism underlying how TNL NADase activity transmits the signal to activate two different EDS1 modules remains unknown.

It was reported that TIR domain in plants hydrolyze nicotinamide adenine dinucleotide in its oxidized form (NAD^+^) to produce v-cADPR (2′ cADPR) (Wan et al. 2019). Recently, plant TIR domain was also found to possess 2′, 3′-cAMP/cGMP synthetases activity by hydrolyzing dsRNA/dsDNA and 2′,3′-cAMP/cGMP are also required for TIR-mediated cell death in plants (Yu et al. 2022). TIR-catalyzed small molecules induced the assembly of Arabidopsis EDS1-SAG101-NRG1 and EDS1-PAD4-ADR1 complexes in insect cells (Yu et al. 2022). To dissect which small molecules activate two different EDS1 signalling nodes, in two recent remarkable papers published in Science, Jijie Chai’s group and partners identified the TIR enzymatic products and disclosed the receptor mechanism for those TIR-catalyzed signaling molecules (Huang et al. 2022; Jia et al. 2022). First, the authors reconstituted the TNL resistosome-induced EDS1-PAD4 association with ADR1 in insect cells. Arabidopsis TNL receptor RPP1, its activating pathogen effector ATR1, EDS1, PAD4 and ADR1-L1 were co-expressed in insect cells and EDS1-PAD4 complex was purified. The purified EDS1-PAD4 complex was expected to bind with RPP1 resistosome-catalyzed products. The extracted small molecules from purified EDS1-PAD4 complex were analyzed by liquid chromatography coupled with high resolution mass spectrometry (LC-HRMS). The chemical structure of identified two compounds was further determined through resolving the crystal structure of purified EDS1-PAD4 complex. Combined the LC-HRMS and structural data, the authors found that the small molecules associated with EDS1-PAD4 protein are ADPR isomer: 2′-(5″-phosphoribosyl)-5′-adenosine monophosphate (pRib-AMP) and 2′-(5″-phosphoribosyl)-5′-adenosine diphosphate (pRib-ADP). By using the similar strategies, the authors also purified EDS1-SAG101 complex in an immune-activated state and identified that the small molecules bound in EDS1-SAG101 complex are ADP-ribosylated ADPR (di-ADPR) and ADP-ribosylated ATP (ADPr-ATP). Interestingly, the NAD^+^ hydrolyzate 2′ cADPR can be catalyzed by TIR to produce di-ADPR or ADPr-ATP. TIR can further hydrolyse di-ADPR or ADPr-ATP to generate pRib-AMP or pRib-ADP (Huang et al. 2022; Jia et al. 2022). These results are consistent with earlier reports that TIR NADase activity is necessary for the TNL-mediated immune signaling (Horsefield et al. 2019; Wan et al. 2019).

The next question is how these small molecules activate two different downstream EDS1 signaling modules. Using biochemical and structural approaches, the authors demonstrated that EDS1-PAD4 is the receptor complex for the pRib-ADP/AMP. The binding of pRib-ADP/AMP allosterically promotes EDS1-PAD4 association with ADR1-L1. In contrast, di-ADPR and ADPr-ATP specifically bind to EDS1-SAG101 and allosterically promote EDS1-SAG101 interaction with NRG1, establishing that EDS1-SAG101 functions as a receptor complex for di-ADPR and ADPr-ATP. Structural analysis reveal specific recognition mechanisms for pRib-ADP/AMP by EDS1-PAD4 or di-ADPR and ADPr-ATP by EDS1-SAG101 (Huang et al. 2022; Jia et al. 2022). Structural data also demonstrated that the binding of small-molecule compounds causes the conformation changes of EDS1-PAD4 or EDS1-SAG101. Compared to the apo-form of PAD4 or SAG101, the EP domain in pRib-ADP bound PAD4 or ADPr-ATP bound SAG101 rotates anti-clockwise about 10 or 15 degrees, respectively. The EDS1 conformation is nearly identical in the apo form and pRib-ADP/ADPr-ATP bound EDS1 (Huang et al. 2022; Jia et al. 2022). Therefore, the conformation changes of EP domain may play critical roles in the specific interactions with two different helper NLRs. Although pRib-ADP and ADPr-ATP are almost completely buried in a pocket formed by EP domains and are unlikely to participate in direct interaction with helper NLRs, whether pRib-ADP and ADPr-ATP can be exposed after EDS1 complex interacts with helper NLRs to activate the helper NLRs remains unknown.

In summary, two remarkable papers published in Science identified the pRib-ADP/AMP and di-ADPR/ADPr-ATP as two types of signaling molecules catalyzed by TIR-containing proteins and revealed that two different small molecules selectively activate the downstream two EDS1-mediated immune branches. Importantly, chemically synthesized pRib-ADP/AMP or di-ADPR/ADPr-ATP can function as second messengers to induce immune signaling (Huang et al. 2022; Jia et al. 2022). Thus, it opens a completely new way to design small molecule chemicals to boost plant innate immunity. EDS1 localizes in both nuclear and cytoplasm. Both subcellular locations are required for EDS1 to confer a complete disease resistance (Garcia et al. 2010). Whether TIR-catalyzed molecules function in EDS1 subcellular localization and whether two different EDS1 signaling complexes enable the transcriptional activation of diverse defense genes in the nuclear need further characterization in the future.

## Data Availability

Not applicable.
